# An optimized sporulation method for the wheat fungal pathogen *Pyrenophora tritici-repentis*

**DOI:** 10.1186/s13007-021-00751-4

**Published:** 2021-05-19

**Authors:** Silke Jacques, Leon Lenzo, Kofi Stevens, Julie Lawrence, Kar-Chun Tan

**Affiliations:** Centre for Crop and Disease Management, Curtin University, Perth, Australia

**Keywords:** Conidia, Sporulation, Tan spot, *Pyrenophora*, Wheat, Plant-pathogen, Plant disease

## Abstract

**Background:**

The necrotrophic fungal pathogen *Pyrenophora tritici-repentis* (*Ptr*) causes tan (syn. yellow) spot of wheat and accounts for significant yield losses worldwide. Understanding the molecular mechanisms of this economically important crop disease is crucial to counteract the yield and quality losses of wheat globally. Substantial progress has been made to comprehend the race structure of this phytopathogen based on its production of necrotrophic effectors and genomic resources of *Ptr.* However, one limitation for studying *Ptr* in a laboratory environment is the difficulty to isolate high spore numbers from vegetative growth with mycelial contamination common. These limitations reduce the experimental tractability of *Ptr*.

**Results:**

Here, we optimized a multitude of parameters and report a sporulation method for *Ptr* that yields robust, high quality and pure spores. Our methodology encompasses simple and reproducible plugging and harvesting techniques, resulting in spore yields up to 1500 fold more than the current sporulation methods and was tested on multiple isolates and races of *Ptr* as well as an additional seven modern Australian *Ptr* isolates. Moreover, this method also increased purity and spore harvest numbers for two closely related fungal pathogens (*Pyrenophora teres* f. *maculata* and f. *teres*) that cause net blotch diseases in barley (*Hordeum vulgare*), highlighting the usability of this optimized sporulation protocol for the wider research community.

**Conclusions:**

Large-scale spore infection and virulence assays are essential for the screening of wheat and barley cultivars and combined with the genetic mapping of these populations allows pinpointing and exploiting sources of host genetic resistance. We anticipate that improvements in spore numbers and purity will further advance research to increase our understanding of the pathogenicity mechanisms of these important fungal pathogens.

**Supplementary Information:**

The online version contains supplementary material available at 10.1186/s13007-021-00751-4.

## Background

Tan (syn. yellow) spot (TS) is an economically important disease of wheat and causes significant crop losses worldwide [1]. The causal agent of TS is the ascomycete fungus *Pyrenophora tritici-repentis* (*Ptr*) which feeds and kills off the host tissue, thereby forming the characteristic necrotic and/or chlorotic spot-like lesions [2]. Yield losses can peak as high as 50% on susceptible varieties and results in global industry impacts with annual losses estimated to exceed US$150 million dollars in Australia alone and TS ranking consistently in the top three most damaging fungal wheat pathogens in the USA [1, 3–5]. Moreover, it has been reported that *Ptr* has also been isolated from other cereal crops such as barley and other grasses [6, 7]. This emphasizes the importance of identifying and understanding the pathogenicity mechanisms between *Ptr* and its hosts as to reduce the damage caused by this economically important plant pathogen.

*Ptr* secretes necrotrophic effectors (NE) during infection to kill the host tissue [8]. In accordance to effector-triggered susceptibility (ETS), the necrosis and/or chlorosis will only manifest itself if the host carries the corresponding susceptibility gene [9, 10]. Thus far three NEs have been characterized in *Ptr*, namely ToxA, ToxB and ToxC [6, 11]. Whereas ToxA and ToxB are small secreted proteins, ToxC is a metabolic effector and each NE induces specific phenotypic disease symptoms [11–13]. ToxA induces dark necrotic lesions occasionally associated with a chlorotic halo, whilst ToxB induces chlorosis as does ToxC, with the latter able to spread the chlorosis through the leaf [14, 15]. These disease symptoms will only effectuate on those wheat lines carrying the dominant susceptibility genes which are *Tsn1*, *Tsc2* and *Tsc1* that interact with ToxA, ToxB and ToxC, respectively [16]. Through the identification of three differential wheat lines, each carrying one of these susceptibility genes, it was now possible to globally classify and distinguish eight races of *Ptr* [17]. The classification of *Ptr* into race 1 to 8 is based on the *Ptr* NE profile and their disease scoring in these three differential wheat lines (Glenlea, 6B662, 6B365) [1]. The molecular knowledge gained within the *Ptr*-wheat pathosystem and the establishment of a global race structure now allows for the screening of host genetic resistance and the development of more effective and sustainable control measures [18].

Besides the progress made in untangling the complex NE regulation [19, 20], valuable genomic resources have been generated of multiple *Ptr* isolates across different races. For example, a high quality *Ptr* reference genome of the Australian race 1 isolate M4 (*ToxA, ToxC*) was assembled and annotated using PacBio sequencing technology [21]. In addition, seven pathogenic *Ptr* isolates, including the race 5 DW5 isolate (*ToxB*) from the USA, were sequenced using Illumina paired-end reads [15]. Along with the ability to genetically transform this fungal pathogen [13, 19], these attributes make *Ptr* an ideal species for virulence studies.

Inoculation experiments with asexual spores (or conidia) form a cornerstone in the research field of many fungal-plant pathosystems. This includes the need to carry out disease resistance screening [18, 22], comparative phenotypic assays [23], isolate maintanence, quantitative trait locus analyses of biparental populations and genome wide association studies of diversity panels [24, 25]. Already in 1977, the effects of substrate, temperature and photoperiod on sporulation of *Ptr* were studied [26]. Odvody and Boosalis [27] reported a sporulation technique which involved carrying out an initial culture on potato-dextrose agar, transferring mycelial plugs onto V8 agar followed by induction of conidiophore production under a light source emitting wavelengths of less than 350 nm, crucial for subsequent conidia production. This method was further simplified by Raymond et al. [28] by including both a PDA and V8 sector in the same petri dish to help induce conidiophore production. It was not until Lamari and Bernier [29] formulated their inoculum production method in 1989 which combined these culture media to a single V8-PDA agar, that this would become a commonly used method to collect *Ptr* conidia. However, this approach remained time-consuming and labour-intensive to harvest sufficient conidia numbers for applications such as infection assays [22]. To overcome this limitation, Dinglasan et al. [22] raised conidia by overlaying infected wheat stubbles on a TS-susceptible wheat variety followed by overhead watering to generate a substantial amount of conidia (2 × 10^5^ conidia in 20 ml of spore suspension) for infection studies. Despite the high inoculum number, the production of conidia using live plants from stubble-borne infection is time intensive and non-axenic, thus run the risk of containing co-infecting fungal pathogens of wheat [30].

In this study, we set out to develop an optimized sporulation protocol using in vitro culturing of *Ptr* that is simple, time-efficient and can consistently generate high spore inoculum numbers, free of mycelial/microbial contaminations. To efficiently harvest the conidia raised through this optimised approach, we devised a simple harvesting strategy by taking advantage of the hydrophobic property of fungal conidia. By adopting these modified approaches, we were able to devise a simple protocol that repeatedly generates three orders of magnitude more conidia per vegetative petri dish (3 × 10^6^) compared to the Lamari and Bernier-based method [29]. Moreover, no mycelial contamination could be observed in the conidial extract. Here, we describe the modifications and optimisations that led to our inoculum production method and evaluate this optimized protocol on a range of *Ptr* isolates. We also tested this improved method on taxonomically related fungal pathogens of barley that causes net form diseases of barley, *Pyrenophora teres* f. *teres* and f. *maculata*, and similarly observed significant improvements in conidia production and purity.

## Results

### Identifying optimal growth conditions

For precise laboratory experiments and large-scale plant infection assays, ideally a simple, short and robust sporulation method that can generate high *Ptr* spore numbers and avoid mycelial contamination would be desired. Currently, the routinely used method described by Lamari and Bernier [29] typically yields between a 1000–3000 spores per vegetative plate, which is relatively low compared to most other fungi [13, 31]. To raise sufficient spore inoculum for wheat bio-assays, numerous petri-dishes are needed for vegetative growth and subsequent conidial generation, thereby significantly increasing space occupancy and labour time. Moreover, using conidia counts as a quantifiable input biomass becomes difficult. In our hands it was difficult to avoid contamination of hyphae during spore harvest. To generate higher yields of *Ptr* spores free of mycelial contamination, we tested a multitude of parameters at each of the different stages involved in sporulation. A focus on five main variables was established, covering the vegetative growth stage, conidiophore and conidia production as well as spore recovery and storage methodologies (Table 1). Within each of these stages, various factors were assayed and the resulting spore numbers were compared to the commonly used sporulation method [29]. These factors included duration of growth, light source, light cycles, temperature, gas exchange, growth and transfer media, plug location and wounding method (Table 1).

**Table 1:** The five stages and corresponding parameters tested for the formulation of an optimized sporulation protocol

Vegetative growth	Conidiophore production	Conidia production	Spore recovery	Purity & storage
Time	Wounding method	Temperature	Release method	Extraction solution
Oxygenation	Plug location	Time	Brush type	Storage medium
Light vs dark incubation	Transfer medium	Dark incubation	Harvest medium	Brush type
Light source	Light source		Plate preparation	Extraction methodology
Growth medium	Time			Glycerol stock
	Temperature			
	Oxygenation			

#### Vegetative growth

Here, we will highlight some of the factors that had a major impact on final spore concentrations and are different to the commonly used method [29]. First, we revisited the earliest published *Ptr* protocol by Odvardy and Boosalis [27] and modified the vegetative growth medium to V8-PDA, as suggested by Lamari and Bernier, but incubated the *Ptr* cultures in a 12 h light: dark regime, rather than continuous dark incubation.

#### Conidiophore and conidia production

**Fig. 1:**
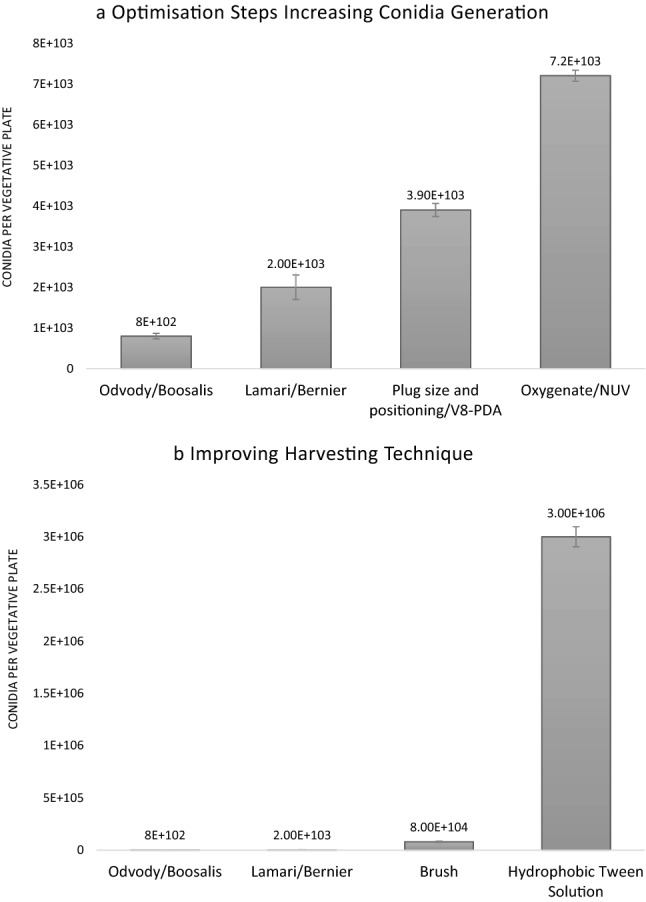
Optimizing multiple parameters to maximize conidia generation. Some of the factors that had a major impact on vegetative growth, condidiophore and conidia generation are highlighted and compared to the traditional methods (**a**). By introducing a paint brush, removing the excess agar and afterwards combining the optimal parameters whilst adding a detergent Tween 20 solution for harvesting, *Ptr* spore numbers were further improved (**b**)

Next, we tested the effect of different sizes of mycelial discs (plugs) as well as the number of transferred discs, the placement of the discs and even the type of media of the transfer plate (V8-PDA; V8; PDA; ½ PDA), all of which resulted in different final spore numbers. The full set of tested values for each parameter is described in Additional file 1: Table S1. The biggest increase was obtained when we transferred 16 small discs (7 mm) to the outer rim of fresh V8-PDA medium (Fig. 1a). Instead of growing these transferred disc plates again for multiple days, we immediately placed these plates in a dark incubator with a specific NUV light (290–400 nm) without any prior flooding or flattening. An important parameter for maximal conidiophore production was the oxygenation of the plates whilst incubated under this NUV irradiation (Fig. 1a). A significant increase in conidiophore and subsequent conidia production was observed when the plates with mycelial *Ptr* discs were incubated without a lid, promoting maximal airflow. Microbial contamination was closely monitored by always including an empty V8-PDA plate as a negative control and vigorous ethanol decontamination of the NUV incubator was proven a sufficient control measure. Additionally, V8-PDA medium can also be supplemented with common antibacterial antibiotics such as kanamycin and streptomycin. Whilst these modifications improved vegetative growth and conidiophore and conidia generation, further improvements were needed to recover these conidia in a clean and efficient manner.

#### Spore recovery

A major focus for developing the harvesting technique was on the increased quantity and purity of spores, which would subsequently lead to improved utility and reproducibility for downstream applications. We split the spore recovery stage further into six smaller categories to allow a step-by-step testing of multiple parameters within the pre-harvest preparation, the choice of flooding solution, the release, extraction, purification and concentrating method (Additional file 1: Table S2). The traditional flooding, scraping or vortexing to dislodge the conidia from the conidiophores usually results in some form of hyphal contamination [13, 14, 27, 29]. Therefore, we opted for a less intrusive method and used a soft paintbrush to release the conidia without disturbing the mycelia (Fig. 1b). An additional step of removing the excess agar around the plugs was also introduced to optimize spore purity. However, using water as our extracting solution was not yielding an increase in *Ptr* spore numbers. Conidia are hydrophobic due to surface proteins such as hydrophobins and adhesins which aid in their dispersal and host immune evasion as well as mediating host adherence [32]. Therefore, we added a detergent (0.02% Tween 20 solution) to gently lift the hydrophobic spores over the mycelia with the brush (Fig. 1b). This avoided the need for flooding and crushing and therefore resulted in pure spore solutions void of any mycelial contamination.

Each of these parameters within the different stages that resulted in a higher number of *Ptr* spores and increased purity were then combined and tested for their additive effects on spore yield. Ultimately, we combined all the top parameters to generate our optimized sporulation protocol that contributed significantly to our qualities of interest.

### An optimised sporulation protocol

**Fig. 2:**
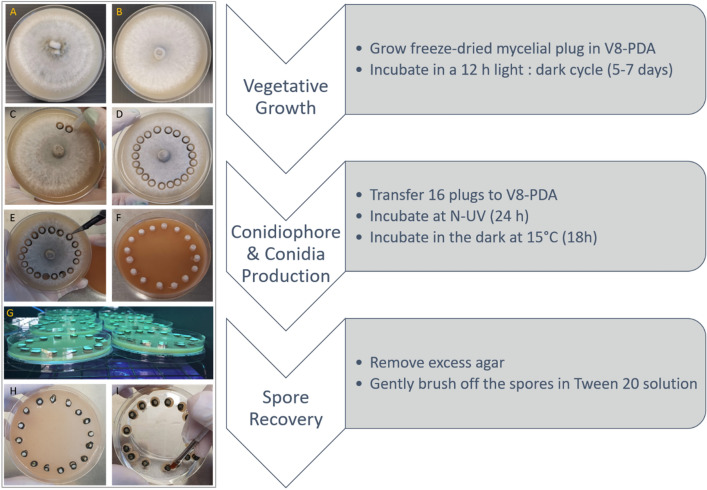
An overview of the optimized sporulation protocol. One-week old vegetative growth V8-PDA plates (**a** = M4; **b** = DW5 *Ptr* isolates) are plugged with the end of a 200 µl pipettip (**c**) about 0.5 cm from the outer rim of the mycelial growth (**d**). A total of 16 plugs are transferred with a sterile blade (**e**) onto a fresh V8-PDA plate and plugs are evenly spaced (5 mm) along the edge of the plate (**f**). Plates are incubated without lids under N-UV light (**g**) for 24 h which induces conidiophore production (**h**). After 18 h incubation in the dark at 15 °C the excessive agar is removed and spores are brushed off in a 0.02% Tween 20 solution (**i**). The flow chart on the right summarizes the three main phases and steps involved in the optimized sporulation protocol

**Fig. 3:**
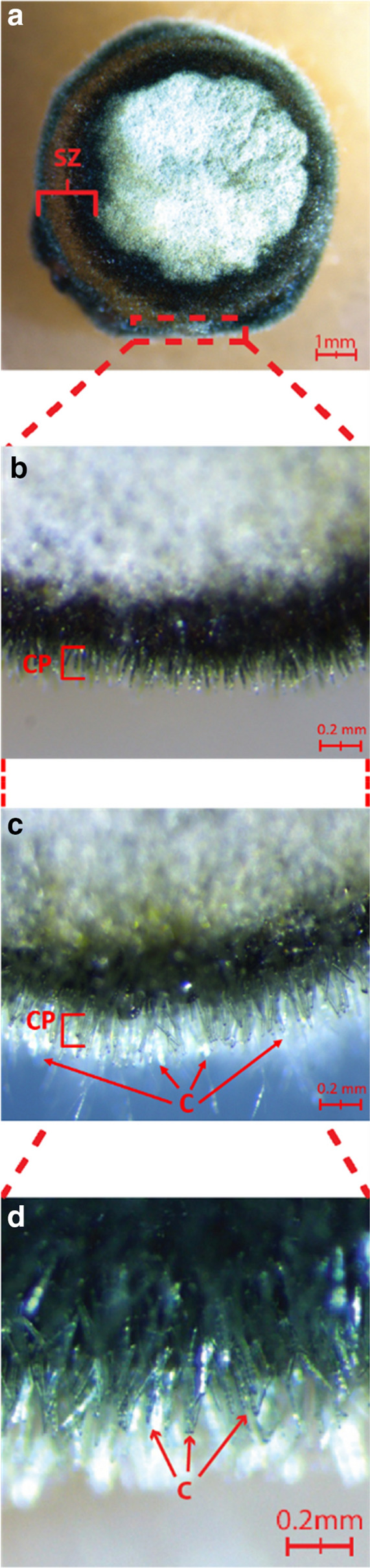
The formation of conidial masses on sporulation plugs. After N-UV irradiation, mycelial plugs are showing a pigmented peripheral sporulation zone (SZ) (**a**). Zooming in shows the production of conidiophores (CP) is causing the blackening of the mycelial edge (**b**). Incubation at 15 °C in the dark for 18 h facilitate dense conidia (**c**) development and maturation (**c**, **d**)

The optimized sporulation protocol was developed using the Australian M4 race 1 reference strain. A step-by-step overview of the protocol is shown in Fig. 2 with the final parameters described in Table 2. The N-UV exposure of the plugged plates (Fig. 2g) results in a darkened rim around the plugs (Fig. 2h) which intensifies after the dark incubation at 15 °C (Fig. 2i). Using a stereoscope to zoom in, the formation of conidiophores (CP) is observed in the darkened ring of the plugs, the so-called sporulation zone (SZ) (Fig. 3a, b). Subsequent dark incubation allows for growth and maturation of the conidia (C) (Fig. 3c, d). The sporulating edge can then be gently brushed off to harvest the spores. This optimized sporulation protocol increased total spore numbers up to 1500 times for the M4 *Ptr* isolate and resulted in an average of 3 × 10^6^ spores per plate (Table 3).

**Table 2:** Growth conditions for conidia production in *Pyrenophora sp.* Columns differentiate growth phases and row describe parameters. All listed conditions were significantly associated with increased conidia production and purity

Parameters	Vegetative	Conidiophore	Conidia	Harvest
Duration	5–7 days	24 h	18 h	–
Light Source	White	N-UV	Darkness	–
Light Cycle	12 h Light/Dark	Continuous	Continuous	–
Temperature	22–24 °C	22–24 °C	15 °C	–
Plate Lid	Sealed	Removed	Loose	–
Media	V8-PDA	V8-PDA	–	0.02% Tween 20
Plug Location	Total Mycelia	–	–	–
Wounding Method	200 µl Tip End	–	–	–
Extraction	–	–	–	Small brush Excess agar removed

**Table 3:** The optimized sporulation protocol tested on *Ptr* strains and *Pyrenophora* species resulting in elevated conidia numbers

Species	Isolate	Race	Year Isolated	Location	Crop	Average Spores Per Plate	Traditional Method	Increase
*Ptr*	M4	1 (ToxA and ToxC)	2009	Meckering (WA)	Wheat	3.00E+06	2.00E+03	**1500 X**
*Ptr*	DW5	5 (ToxB)	1998	North Dakota (USA)	Wheat	1.79E+06	1.50E+03	**1194 X**
*Ptr*	15 FRG 233	1 (ToxA and ToxC)	2015	Esperance (WA)	Wheat	1.37E+06		
	15 FRG 239	1 (ToxA and ToxC)	2015	Yarrawonga (VIC)	Wheat	3.30E+06		
	15 FRG 022	1 (ToxA and ToxC)	2015	Dandaragan (WA)	Barley	1.75E+06		
	17 FRG 118	1 (ToxA and ToxC)	2017	Wagin (WA)	Wheat	2.47E+06		
	15 FRG 230	1 (ToxA and ToxC)	2015	Cunderdin (WA)	Wheat	3.00E+06		
	17 FRG 135	1 (ToxA and ToxC)	2017	Cowra (NSW)	Wheat	2.64E+06		
	16 FRG 175	1 (ToxA and ToxC)	2016	Wunkar (SA)	Wheat	1.15E+06		
*Ptt*	W1-1	"Beecher avirulent"	2009	Wongan Hills (WA)	Barley	1.00E+06	1.00E+03	**1000 X**
*Ptm*	SG1	unknown	1996	Badgingarra (WA)	Barley	8.10E+05	5.00E+03	**162 X**

Next, we wanted to validate this protocol for other Australian *Ptr* isolates and included seven isolates that were collected from the field across four different states (Additional file 1: Figure S1). All isolates were taken from wheat with the exception of one Western Australian sample (yellow), which was isolated from barley (Additional file 1: Figure S1). For all seven *Ptr* isolates, we were able to isolate over a million spores per vegetative growth plate, irrespective of the year of isolation, the crop it was isolated from or the location (Table 3). One isolate from Victoria consecutively yielded the highest number of *Ptr* conidia (3.3 × 10^6^). Taken together, these numbers support our method as a robust and reliable sporulation protocol for Australian *Ptr* isolates.

We aimed to develop a protocol for the broader *Ptr* community so next we tested the ToxB-producing DW5 race 5 strain from the USA, which also has excellent genomic resources available, making it the ideal testing candidate [33]. Originating from a single vegetative plate, the average number of *Ptr* spores was repeatedly exceeding one million compared to an average of 1500 spores that we were able to isolate using the Lamari and Bernier method [29]. This more than 1000-fold increase in conidia numbers further supports the universal nature of this method for the vast majority of *Ptr* isolates since collectively, the M4 and DW5 strains produce all three known effector molecules (*ToxA*, *ToxB* and *ToxC*) thus far characterized in *Ptr*.

### *Ptr* spore inoculum is hyphal-free, viable and highly infectious

Reliable and consistent quantification of input material is crucial to perform comparative wheat infection studies with *Ptr*. Therefore, mycelial contamination should be avoided so spore counts can be a robust measure for input biomass.

#### Hyphal-free spore inoculum

**Fig. 4:**
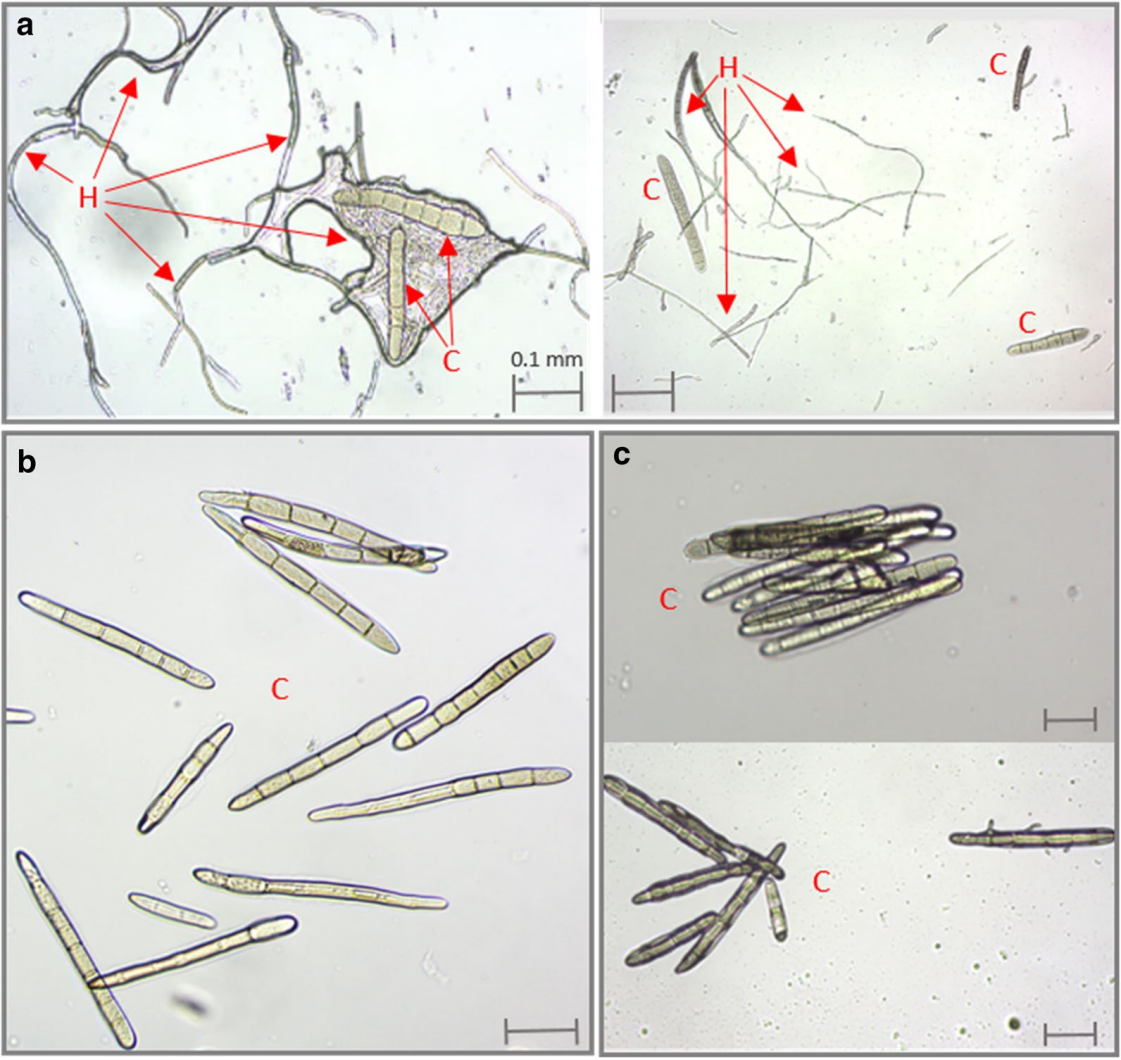
Mycelial contamination was avoided with the optimized sporulation method. Microscopy images show the mycelial impurities (H = hyphae) of the harvested conidia (C) with the traditional Lamari and Bernier sporulation method (**a**). Using our optimized protocol, mycelial strands can be avoided and pure DW5 (**b**) and M4 (**c**) *Ptr* spores are obtained. Scale represents 0.1 mm on all images

With the traditionally used method [29], mycelial contamination was difficult to avoid as can be seen in panel A of Fig. 4. With our optimized method, very pure spore solutions are obtained with no mycelia detected in both the DW5 (Fig. 4b) and M4 (Fig. 4c) harvested spore solutions. The lack of mycelial impurities was consistent with every spore isolation and as such, we can confirm our optimized sporulation protocol yields high and pure conidia.

#### Viable spore inoculum

**Fig. 5:**
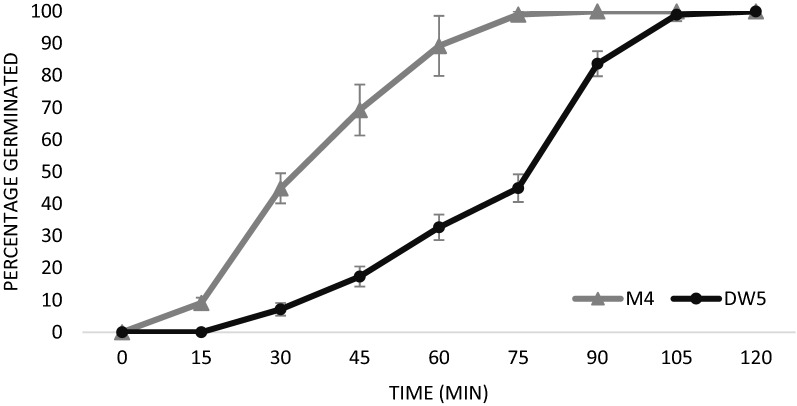
Conidial germination rate of *Ptr* M4 and DW5. Viability of harvested spores was followed every 15 min under the microscope by counting the spores that formed a protruding germination tube. Within 30 min, M4 reached 44.9% germination rate whilst for DW5 this took 75 min. All M4 spores germinated within 1.5 h of harvesting whereas 100% germination rate of DW5 was achieved within 2 h

Part of the optimised protocol required the use of the detergent Tween 20 to liberate conidia from the conidiophores. To test that the inclusion of Tween 20 does not reduce the viability of *Ptr*, we then examined harvested conidia by determining their germination rate through the formation of germ tubes. *Ptr* M4 and DW5 isolates were used and germination lagged in the DW5 spores. Whilst 45% of the M4 spores germinated within 30 min, DW5 only reached this germination percentage after 75 min (Fig. 5). Within two hours, all the spores formed a germination tube, thereby reaching 100% germination rate.

#### Infectious spore inoculum

**Fig. 6:**
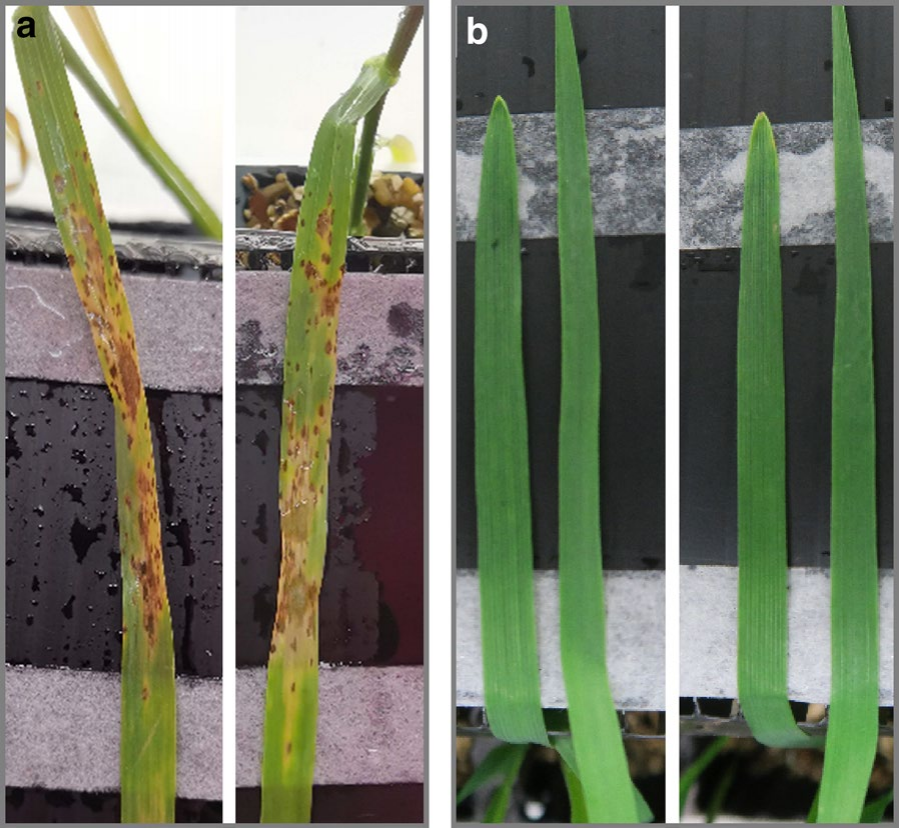
*Ptr* spore inoculum obtained from the improved protocol remained infectious on wheat. An attached leaf infection assay with viable M4 *Ptr* spores (1 × 10^5^/ml) demonstrated typical tan spot symptoms (**a**) whereas the negative controls sprayed with 0.02% Tween 20 solution did not (**b**)

To verify whether the *Ptr* conidia can infect host tissue, we performed attached wheat leaf bioassays*.* Within 5 days after spraying the isolated *Ptr* spore inoculum on susceptible 2 week-old wheat leaves, a clear disease phenotype was visible with the characteristic necrotic and chlorotic lesions of tan spot (Fig. 6a). This distinct phenotype was not visible in the negative control, where wheat leaves were sprayed in a similar manner with a 0.02% Tween 20 solution (Fig. 6b). These figures are representative pictures as we performed *Ptr* spore infections repeatedly covering a range of *Ptr* spore inoculum concentrations, each time yielding good infection and leading to phenotypic chlorotic and necrotic lesions on the wheat leaf.

Taken together, the optimized protocol generates highly concentrated *Ptr* inoculums free of hyphal contamination. The generated spores are viable and highly infectious, thereby enabling large-scale infection assays with pure *Ptr* spore suspensions whose counts can be used as a reliable biomass input measurement.

### Increase of conidia numbers in other fungal phytopathogens

In addition, to investigate the feasibility of this method for additional fungal plant pathogens in the *Pyrenophora* family, we also tested the sporulation protocol on two other important phytopathogens, namely *Pyrenophora teres* f. *teres* (*Ptt*) and *Pyrenophora teres* f. *maculata* (*Ptm*) causing net form net blotch and spot form net blotch in barley, respectively. Compared to their traditional isolation methods, *Ptt* spore numbers were increased a 1000-fold whereas *Ptm* spores increased over 160 times (Table 3).

Overall, our modified inoculum production protocol was validated on two *Ptr* strains belonging to different races, a total of seven Australian field isolates as well as two additional *Pyrenophora* species, namely *Ptt* and *Ptm*. Using our method, all isolates significantly increased conidia generation, which resulted in higher numbers of purified spore inoculum.

## Discussion

This study presents a sporulation method that permits high, pure and infectious spore isolations of important fungal necrotrophic phytopathogens of wheat and barley. This sporulation protocol details every optimized parameter and introduces new ways of harvesting conidia to avoid mycelial contamination. Moreover, this protocol can be completed within just seven days and this includes the vegetative growth stage of the pathogen. Spore inoculum levels were increased significantly (> 1000-fold) for both the M4 and DW5 *Ptr* reference strains which collectively represent the whole suite of effector molecules thus far characterized in *Ptr*. A steep increase in spore numbers was also obtained for seven modern *Ptr* field isolates as well as for two related fungal pathogens causing net blotch disease in barley (*Ptt* and *Ptm*). This further highlight we provided a useful tool to accelerate *Pyrenophora* research aiming to better understand and manage the impact of these globally devastating fungal pathogens.

To dissect the molecular mechanisms of *Ptr* pathogenicity and virulence or to screen for novel sources of resistance, large spore inoculums are typically required. We succeeded in developing a sporulation protocol that minimizes space occupancy and time and maximizes spore numbers absent from mycelial contamination. Currently, the most common and widely used *Ptr* sporulation method dates back to 1989 and usually only yields between 1000 to 3000 spores per vegetative growth plate [29]. Hence, a sizeable number of vegetative plates needs to be cultured for a single experiment, making it time-consuming and labour-intensive. Therefore, we set out to formulate an optimized sporulation protocol for *Ptr* by testing and adapting previously reported sporulation methods and including new techniques. We focused on five main stages during sporulation and assayed a multitude of parameters within each of these pillars (Table 1, Fig. 1). We also included some of the earlier described protocols than the currently used method as minor tweaking differences showed to have an impact on final spore numbers. Different growth and transfer media were tested (Additional file 1: Table S1) as Odvody and Boosalis (1982) found that large numbers of conidia could be obtained by first culturing the fungus on potato dextrose agar (PDA) and then transferring disks from the colony margins onto a modified V8 agar medium [27]. In the commonly used method, single conidia were isolated from infected leaf samples taking up an additional 48 h [29]. Thereafter, plugs of a 4 to 8 day culture were transferred to single plates and further incubated for another 5 days before flattening the mycelia and another 48 h incubation (fluorescent light and dark) [29]. In total, this method can take up to 17 days to carry out. The optimised protocol devised in this study significantly reduces handling time and only takes between 7 and 9 days to complete. A summarizing comparison (Table 4) highlights the simplicity of our method by greatly reducing the number of steps whilst generating higher conidial output (Summary Table 4). In agreement with the commonly used method, the combination of V8 and PDA into a single medium increases conidia production compared to the method of Raymond et al. (1985) [28] (Fig. 1a). Rather than continuous darkness [29] or continues light conditions [28] for vegetative growth, we opted for a 12 h light: dark regime. As mycelia are also able to infect host tissue, mycelial contamination of spore inoculums could skew pathogenicity results. It was shown that scraping inoculum from agar surfaces results in an inoculum where only 67% of total propagules were conidia, the other 33% included hyphal fragments and conidiophores [34]. Hence why we focused on the harvesting technique to yield high and pure spore solutions. Six parameters were identified and the biggest novelties were the removal of excess agar around the discs, the introduction of a particular sized paintbrush to gently release the conidia and the use of a Tween 20 solution (Additional file 1: Table S2, Fig. 1c) rather than the classical dislodging via vortexing, scraping or flooding. The non-ionic surfactant Tween 20 helps lower the surface tension between the liquid harvest solution and the solid fungal conidia attached to their conidiophores. The addition of Tween 20 was proven crucial to release the conidia since the latter are hydrophobic due to their conidial surface proteins, which play an important role in germination, stress resistance, adhesion and virulence [32]. The effect of Tween 20 on germination and vegetative growth has been studied in other fungi, was proven compatible with germination studies at lower surfactant concentrations and can enhance the uptake and transport of conidia [35, 36]. By combining all the optimal and improved parameters involved in sporulation, we delivered an optimized sporulation protocol (Table 2, Fig. 2).

**Table 4:** A method comparison of the optimized conidia production in Pyrenophora sp. (this study) to the Lamari and Bernier 29 protocol

	Lamari and Bernier	This study
Vegetative Growth	Grow stock culture from infected leaf sample in V8-PDA (4–8 days)	Grow freeze dried mycelial plug in V8-PDA (5–7 days)
Conidiophore Production	Transfer and Incubate in the dark single mycelial plugs on V8-PDA (3–5 days)	Transfer 16 plugs to V8-PDA and incubate at N-UV (24 h)
	Flood culture with water and flatten mycelia	
	Decant water and incubate in light at RT (18–24 h)	
	Decant water and incubate in light at RT (18–24 h)	
Conidia Production	Subsequent dark incubation at 15 °C (18–24 h)	Subsequent dark incubation at 15 °C (18 h)
Spore Recovery	Flood plate with water and dislodge conidia with wire loop	Remove excess agar and liberate conidia with fine paint brush in a Tween 20 solution
	Two to three additional water rinses	Spin down (5 min)
	Incubate spore suspension at 5 °C (10–15 min)	
	Decant excess water and add Tween 20	

*Ptr* isolates are divided into races based on their effector profile [37]. We compared the race 1 isolate M4 and the race 5 isolate DW5 to ensure that our protocol can be reproduced on genetically distinct pathotypes. An additional seven Australian isolates were included to compare spore yields. These isolates have a broad geographical distribution and were collected from 4 different states (Additional file 1: Figure S1). Interestingly, one of the *Ptr* isolates was extracted from barley in Western Australia further highlighting the need for tan spot control solutions as wheat is no longer the only host of *Ptr* [7]. Two other damaging fungal pathogens causing net blotch in barley (*Ptt* and *Ptm*) were included in this study to assess the usability of this protocol across related fungal plant pathogens. All isolates produced conidiophores after N-UV exposure, which is visible as a blackened outer rim on the mycelial discs (Fig. 2). Whereas Lamari and Bernier (29) incubate the discs at daylight, Odvody and Boosalis discuss in depth this incubation step and conclude light sources emitting wavelengths less than 350 nm are best to induce conidiophore formation [27, 29]. Near ultraviolet radiation has been shown to initiate or increase sporulation of more than 30 diverse species of fungi [38]. Similar to the devastating fungus that causes brown spot disease in rice (*Cochliobolus miyabeanus*) a subsequent dark period incubation is needed for *Ptr* sporulation [39]. Dark incubation at 15 °C allows for the development of the *Ptr* conidia which is seen as white translucent specks at the end of the conidiophores (Fig. 3c, d). For all isolates tested, the optimized protocol generated higher number of harvested conidia per vegetative plate with the highest increase (1500x) for *Ptr* M4 (Table 3). With the exception of the *Ptm* isolate (8 × 10^5^) all isolates now produced on average a minimum of one million spores per plate, which is a welcome improvement to the traditional yields.

For pathogenicity assays, precise, uniform and high yielding spore inoculum free of mycelial contamination is desired. Microscopy shows that the spore solutions of M4 and DW5 are free of mycelia (Fig. 4b, c) thereby providing a pure inoculum where spore count can be used as a reliable measure for input biomass. Furthermore, both the M4 and DW5 isolates reached 100% germination rate within 120 min of harvesting the conidia (Fig. 5). Tan spot disease developed within 5 days in wheat leaves sprayed with harvested *Ptr* spore solutions with the development of the typical necrotic/chlorotic lesions (Fig. 6). Providing higher spore numbers free of mycelial contamination can further advance our understanding of the pathogenicity mechanisms of these important fungal pathogens.

## Conclusions

By testing and adapting previously reported sporulation methods and including new techniques, we delivered an optimized sporulation method, which can now be implemented as a fast and easy tool to expand our current understanding of the molecular basis of fungal plant infection and pathogenicity. Delivering uniform, precise and reproducible conidia inoculum in a fast and efficient manner allows for standardized and quantifiable large-scale pathogenicity studies. The latter is crucial in the race to develop viable and sustainable control solutions to minimize the devastating impact of these global fungal plant pathogens on crops.

## Methods

### Fungal strains

Isolates were inoculated from mycelial stock onto 90 mm polystyrene petri dishes (Thermofisher, MA, USA) filled with V8-PDA and maintained under a white light on a 12 h light: dark cycle for 5 to 7 days at 22–24 °C. V8-PDA contains 15 g/l agar, 10 g/l Potato Dextrose Agar, 150 mL/l V8 Juice and 3 g/l CaCO_3_. *Ptr* race 1 isolate M4 was isolated from tan spot infected wheat leave samples collected in Meckering (Western Australia) in 2009 [13]. The *Ptr* race 5 DW5 isolate was collected from North Dakota (USA) in 1998 [40]. M4 and DW5 are sequenced reference isolates [21]. The local Australian *Ptr* isolates were collected from four different states, namely one isolate from South Australia (16 FRG 175), four isolates from Western Australia (15 FRG 233, 15 FRG 022, 17 FRG 118, 15 FRG 230), one isolate from New South Wales (17 FRG 135) and one isolate from Victoria (15 FRG 239). These were collected over multiple years ranging from 2015 till 2017 and are all race 1 isolates (containing Tox A and Tox C effectors) (Table 3). The barley phytopathogen isolate W1-1 is a *Pyrenophora teres* f. *teres* (*Ptt*) strain collected from the Wongan Hills in Western Australia in 2009 whilst SG1 is a *Pyrenophora teres* f. *maculata* (*Ptm*) isolate from Badgingarra (WA) collected in 1996 [41].

### Vegatative growth

Isolates were inoculated from mycelial stock, stored at − 80 °C as freeze dried plugs, and single plugs were placed onto 90 mm polystyrene petri dishes (Thermofisher, MA, USA) filled with V8-PDA (15 g/l agar, 10 g/l Potato Dextrose Agar, 150 mL/l V8 Juice and 3 g/l CaCO_3_). Vegetative growth was maintained under a white light on a 12 h light: dark cycle for 5 to 7 days at 22–24 °C until mycelia were nearing the edge of the petri dish (between 70 and 80 mm in diameter).

### Conidiophore and conidia generation

After 5–7 days of vegetative growth, plugs were cut by perforating the mycelia and agar with the wide end of a sterile 200 μl pipette tip (Thermofisher, MA, USA). A single vegetative plate yields approximately 48 plugs and the bevelled edge of the tip maximises mycelial wounding which in turn promotes conidiophore formation. Sixteen plugs were then transferred to a fresh V8-PDA plate and arranged in an evenly spaced ring around its edge approximately 5 mm apart per fresh plate, giving a ratio of 1 vegetative: 3 sporulation plates. These sporulation plates were immediately transferred to an incubator at 22–26 °C, and irradiated for 24 h under a continuous near-ultraviolet (N-UV) light source emitting between 290 and 400 nm wavelength (Actinic BL TL-D, Phillips, Amsterdam, NL). Lids were removed from the petri dishes to maximise oxygen flow and the N-UV irradiation will result in the development of conidiophores in the form of a thick black ring around the plugs outer face, which can be confirmed through stereoscopic analysis. Once conidiophores were visually detected, lids were replaced, though not sealed, and incubated in the dark at 15 °C for 18 h, which induces conidia formation.

### Conidia harvest

Initially, plates were checked for conidial development under a stereoscope. Once spore maturity was confirmed, excess agar was removed from the plate by using a 14 mm cork borer to cut secondary plugs around the sporulating plugs, being careful not to touch any spores during excision. Following excision of excess agar, the remaining agar plugs were flooded with 5 ml of 0.02% Tween 20 (Merck, New Jersey, USA). To maximise conidial release, the petri dish was tilted to achieve complete submersion of a single plug and the surface and edges of the primary submerged plug brushed in a circular motion with a WestArt 663 Sable Flat No.4 paintbrush. Brushing continued until the air trapped by the hydrophobic conidia was released and then repeated on each subsequent plug in the same manner, ensuring submersion was maintained at each step.

Once all plugs were brushed, all remaining agar and mycelia was removed from the dish leaving only the spore solution, which was then poured into a 50 ml Falcon tube (Thermofisher, MA, USA). Spore suspension was spun at 211* g* for 5 min and left to sit at room temperature until all conidia are settled before removing the pellet with a 1 ml pipette with the tip aperture cut to a diameter 2 mm to ensure easier pipetting of conidial masses.

### Conidia counts and viability assays

Spore solutions were quantified by streaking out 3 × 10 µl of spore solution on a superfrost glass microscopy slide (Thermo Scientific, MA, USA) and counting manually with a counter clicker under a Nikon eclipse E200 microscope (10X magnification). To assess spore viability, the germination rate of three diluted spore suspensions, each containing 35 spores, was followed over time until 100% germination rate was reached. Germination was counted under the Nikon eclipse E200 microscope (4X magnitude) where a positive count equals a spore with a protruding germination tube.

### Infection assays

For *Ptr* bioassays, the second leaf of 14-day-old wheat seedlings (cultivar Axe–rated as TS sensitive) was attached, adaxial side up, to corrugated sheeting using microporous surgical tape (Liv-Pore, Mascot, Australia). Leaves were inoculated with an evenly sprayed spore suspension at a concentration of 1 × 10^5^ spores/ml in 0.02% (v/v) Tween 20 using the head from a 0.5 Trigger Sprayer (Nylex, Doncaster, Aus). As a negative control, leaves were attached and sprayed with a 0.02% (v/v) Tween 20 solution as described above. Leaves were allowed to dry before being released from the micropore tape and the wheat seedlings incubated in a controlled growth chamber (CMP6050 Conviron, Controlled Environments Limited, Winnipeg, Canada) under a 12 h light period at 90% relative humidity and 22 °C with regular watering intervals every 4 h. Lesions typically start to form from 3 days post inoculation.

## Supplementary Information

**Additional file 1: Figure S1. Collection sites of seven Australian *Ptr* isolates from the field.** Each collection site is depicted as a grain pictogram with the different colours representing isolates from different states. The exception is Western Australia, where the three blue *Ptr* strains were isolated from wheat whilst the yellow site represents a *Ptr* strain isolated from barley. All other *Ptr* strains were isolated from wheat. This figure was created using Map Data © 2021 Google. **Table S1. Growth conditions tested for conidia production in *Ptr.* Table S2. All harvesting parameters tested for purity and concentration in *Ptr*** Optimal values are underlined.

## Data Availability

All data generated or analysed during this study are included in this published article (and its Additional file 1).
