# Supplementary material for: An optimized sporulation method for the wheat fungal pathogen Pyrenophora tritici-repentis
Source: Plant Methods. 2021 May 19;17:52. doi: 10.1186/s13007-021-00751-4 (PMC8136220; doi:10.1186/s13007-021-00751-4)
Supplement: Supplementary file 1 — Additional file 1: Figure S1. Collection sites of seven Australian Ptr isolates from the field. Each collection site is depicted as a grain pictogram with the different colours representing isolates from different states. The exception is Western Australia, where the three blue Ptr strains were isolated from wheat whilst the yellow site represents a Ptr strain isolated from barley. All other Ptr strains were isolated from wheat. This figure was created using Map Data © 2021 Google. Table S1. Growth conditions tested for conidia production in Ptr. Table S2. All harvesting parameters tested for purity and concentration in Ptr Optimal values are underlined. [file 13007_2021_751_MOESM1_ESM.docx]

**Additional file 1**

**Figure S1. Collection sites of seven Australian *Ptr* isolates from the field**

Each collection site is depicted as a grain pictogram with the different colours representing isolates from different states. The exception is Western Australia, where the three blue *Ptr* strains were isolated from wheat whilst the yellow site represents a *Ptr* strain isolated from barley. All other *Ptr* strains were isolated from wheat. This figure was created using Map Data © 2021 Google.

**
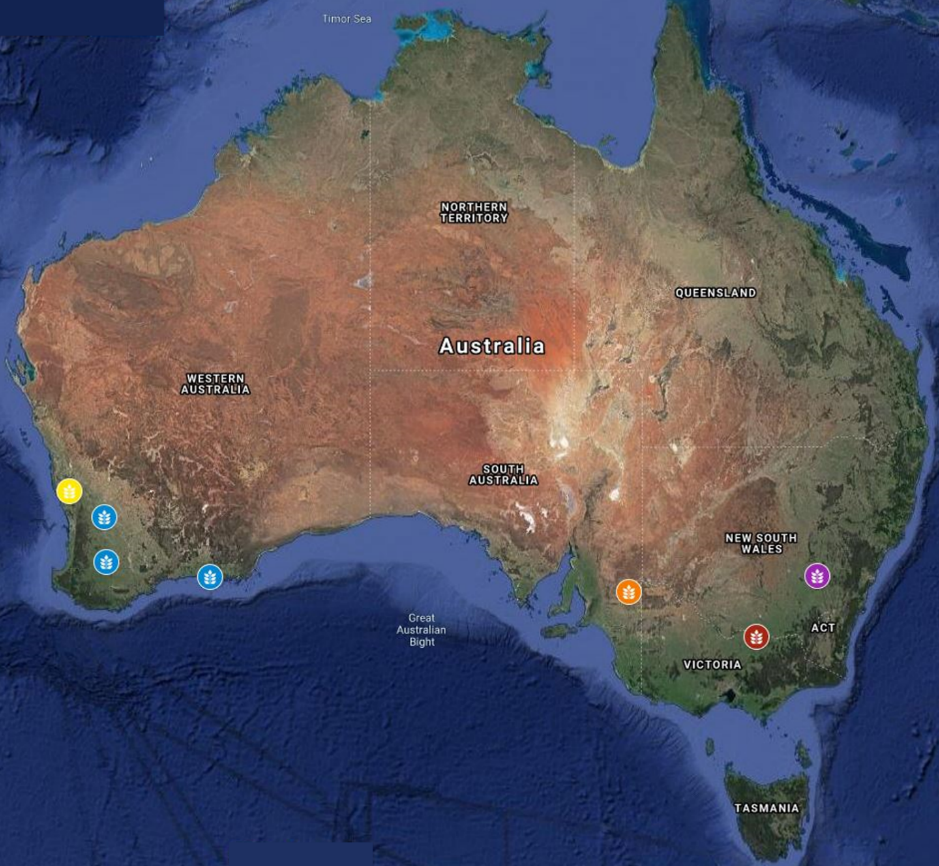
**

**Table S1. Growth conditions tested for conidia production in *Ptr***

| **Parameters** | **Vegetative** | **Conidiophore** | **Conidia** |
| --- | --- | --- | --- |
| Duration | 3, 5, 7, 10, 12, 14 days | 6, 12, 18, 24, 48 h | 6, 12, 18, 24, 48 h |
| Light Source | White, N-UV, UV-B, BLB, Darkness | White, N-UV, UV-B, BLB, Darkness | White, N-UV, Darkness |
| Light Cycle | 12h Light/Dark, Continuous | 12h Light/Dark, Continuous | 12h Light/Dark, Continuous |
| Temperature | 22-26C | 22-26C | 15-24C |
| Plate Lid | Sealed, Removed, Loose | Sealed, Removed, Loose | Sealed, Removed, Loose |
| Growth Media | V8-PDA, ½ PDA, TWA | V8-PDA, ½ PDA, TWA | - |
| Plug Location | Growing Edge, Total | - | - |
| Wounding | 200ul Tip End, 1ml Tip End, Cork Borers | - | - |

**Table S2. All harvesting parameters tested for purity and concentration in *Ptr***Optimal values are underlined.

| **Stages** | **Tested parameters** |
| --- | --- |
| Preparation | None, Remove Excess Agar |
| Solution | Gelatin at 5 %, 0.5 %, 0.05 %, Tween 20 at 2 %, 0. 2%, 0.02 %, H_2_O |
| Release | None, Tip Scraping, Scalpel Scraping, Vortexing, Shaker Rack, Centrifuge, Large Brush, Small Brush |
| Extraction | Regular Pipetting, Pouring, Pipetting with a cut tip, direct pour followed by washing with a cut tip |
| Purification | None, Gauze, cheesecloth, sorbitol gradient, 4000 rpm spin, 1000 rpm spin, |
| Concentration | None, 4000 rpm spin, 1000 rpm spin |
